# Draft Genome Sequences of Two *Streptococcus pneumoniae* Serotype 19F Sequence Type 271 Clinical Isolates with Low- and High-Level Cefotaxime Resistance

**DOI:** 10.1128/genomeA.00605-15

**Published:** 2015-06-11

**Authors:** Margaret Ip, Helen Ma, Carmen Li, Stephen Tsui, Haokui Zhou

**Affiliations:** a Department of Microbiology, Faculty of Medicine, Chinese University of Hong Kong, Hong Kong SAR; b School of Biomedical Science, Chinese University of Hong Kong, Hong Kong SAR

## Abstract

We report here the draft genomes of two pneumococcal isolates in Hong Kong, CU_SPNE1_05 and CU_SPNE32_06. Strain CU_SPNE1_05 had a cefotaxime MIC of 1 µg/ml, and CU_SPNE32_06 had an MIC of 32 µg/ml. Both strains belong to the multidrug-resistant serogroup 19, sequence type 271 (clonal complex 3200/271).

## GENOME ANNOUNCEMENT

*Streptococcus pneumoniae* serogroup 19 multilocus sequence type 271 (ST271) is a multidrug-resistant (MDR) clone and belongs to clonal complex CC 320/271 (1). This clone has been implicated in both invasive pneumococcal disease (IPD) and noninvasive pneumococcal disease in Asia (2–4). In mainland China, 43.8% of IPD cases were reported to be due to serotype 19 during 2005 to 2011, which included ST271 (4, 5).

The Hong Kong Special Administrative Region introduced the childhood pneumococcal immunization program in 2009 with pneumococcal conjugate vaccines (PCV) PCV7, PCV10, and PCV13. Among the serotypes included in PCV7, serotype 19F was the least likely to evoke a protective immune response, according to both vaccine efficacy trials and *in vivo* studies (6, 7). Therefore, despite the decrease in carriage and invasive disease from serotype 19F, the reemergence of the disease is anticipated due to waning of the antibody response over time (8). During this period, an increasing percentage of resistance to a third-generation cephalosporin, cefotaxime, was also observed in *S. pneumoniae* serotype 19F (1).

*S. pneumoniae* strain CU_SPNE1_05 was isolated from a sputum sample from a 90-year-old male in 2005, and strain CU_SPNE32_06 was isolated from a sputum sample from a 52-year-old male in 2006 in a university teaching hospital in Hong Kong. Both strains belonged to serotype 19F and ST271 (http://spneumoniae.mlst.net). Both CU_SPNE1_05 and CU_SPNE32_06 were multidrug resistant, with resistance to lincomycin, erythromycin, and tetracycline and to chloramphenicol and linezolid (9). CU_SPNE32_06 was sensitive to fluoroquinolones but resistant to penicillin and cefotaxime (CTX), while CU_SPNE1_05 was sensitive to fluoroquinolones. The CTX MICs of CU_SPNE1_05 and CU_SPNE32_06 had a 32-fold difference and were 1 µg/ml and 32 µg/ml, respectively.

The genomes were sequenced using Illumina HiSeq platform (90-bp paired-end reads). Genome assembly was performed with metAMOS pipeline (version 1.5rc3, using the iMetAMOS automated ensemble assembly workflow). Prokka (version 1.11) was used for genome annotation, including open reading frame (ORF) finding, RNA gene prediction, and gene function annotation. The genome of CU_SPNE1_05 was assembled into 68 contigs (≥500 bp), with a total length of 2,038,146 bp (sequenced to ~11 million reads with ~400× coverage). The predicted genes from this genome include 1,998 coding sequences (CDSs), 3 rRNAs, and 24 tRNAs. For CU_SPNE32_06, 88 contigs (≥500 bp) were assembled, with a total length of 2,040,265 bp (sequenced to ~15 million reads with ~600× coverage). The predicted genes from the genome include 1,977 CDSs, 3 rRNAs, and 40 tRNAs.

Further studies are under way with the two genomes to understand the evolution of CTX resistance in *S. pneumoniae*.

### Nucleotide sequence accession numbers.

The draft genome sequences of CU_SPNE1_05 and CU_SPNE32_06 have been deposited in the NCBI database under the GenBank accession numbers LCSI00000000 and LCSJ00000000, respectively.
